# Lecture upon Sulphur

**Published:** 1887-11

**Authors:** J. T. Kent


### LECTURE UPON SULPHUR.

PROFESSOR J. T. KENT, A. M., M. D.

(Continued from page 377.)

Fullness in stomach after eating but little (Lyc., Chin., Carb. v.).

Lyc. leads in complaints causing flatulency. Sulph. has the same condition.

Many physicians prescribe Lyc. upon this one symptom: "Every little mouthful makes her feel as if she had eaten a full meal."

Sulph. is a great remedy in the nausea and vomiting during pregnancy, occurring either in the morning or evening. Many of these cases of vomiting are very persistent and troublesome; but when you find it accompanied by the all-gone feeling at eleven A. M., can't wait for her dinner, sour eructations from one to three hours after eating, burning in soles, on head, *agg.* by bathing, you have a pretty good Sulph. case.

You will need to know a great deal of Sep., Puls., Ars., Carb.-veg. in this vomiting of pregnancy. Sep. will be indicated in the vomiting of milky water, with the concomitant of bearing down or funneling, as if the whole viscera of the pelvis were coming into the world, and with a feeling of hunger and emptiness that is almost constant, stomach symptoms relieved by eating, constipation, etc. Carb.-veg is indicated when there is a great amount of belching, belching which gives relief, and also the passing of a great amount of flatus, which gives relief.

Under Sulph. the book says: "Eructations, generally empty, and taste of food," "sour after eating." As soon as she presses upon the stomach there is regurgitation of sour food. Sour, watery vomiting, especially morning and evening; stomach sensitive to touch, *agg.* in the morning. Give the "rugged philosopher" these symptoms, with the all-gone hungry feeling at eleven A. M. It finishes the picture.

A characteristic feature of Sulph. is a *sharp* appetite for any kind of wholesome food, but *at the sight* of food the appetite gives place to disgust.

You must not confound this with Colch. Colch. has no appetite at all, together with disgust at the sight, smell, or even thought of food.

Bry. has a desire for different foods which, when brought to him, are rejected. Rejects the oysters which but a short time before he fancied he would like or had even called for. You will find nausea like that of Colch. in typhoid fevers, in most fevers the patients often going ten days without food.

Sulph. has also vomiting coming on every evening at six o'clock. Clinical.

Sulph. produces great changes of the tissues of the lungs, the liver, and the spleen. It is of wonderful service in enlargement and induration of the liver and spleen. It goes to the bottom of the malarial diathesis, which is the underlying feature of an enlarged spleen. Chin., Nux v., have symptoms relating to enlargement of the spleen, and often cure it. Polymnia urudalia (bear's foot) is a wonderful remedy in enlargement of the spleen, with paucity of symptoms. It has been a great remedy in the South and Southwest for that condition, with malarial cause, hypertrophy of the spleen, ague cake.

Sulph. is a great remedy in flatus. The abdomen is filled with flatus, causing a constant tympanitic condition, bloated abdomen, knotted, incarcerated flatus, spasmodic condition of the intestines, great rumbling in the bowels, painful sensation, as if internally raw and sore, painful swelling of inguinal glands, all in keeping with the general conditions of the remedy. You can see how, like Lyc. and Chin., it easily runs into stomach and bowel troubles. Chin. especially produces incarcerated flatus, with belching, belching, belching, which gives no relief.

Similar to Lyc., opposed to Carb.-v., which finds relief by belching.

Another marked characteristic is that, in spite of extreme care, the odor of the stool follows him for hours.

Cholera (driving out of bed in the morning), cholera Asiatic, cholera morbus, diarrhœa or dysenteria—don't forget Sulph. You will find all varieties of stool, large or small, diarrhœa or dysentery. It has, like *Merc.*, a desire to remain at stool, a never-get-done sort of feeling, with burning in the anus almost like hot coals, caused by the excoriation of the mucous membrane. Passing of blood with stool in chronic bleeding hemorrhoids. Hemorrhoids blue and burning—Phos. ac. Lancinating pains from the anus upward, especially after stool. Pulsating pains in the anus all day. Lach. has throbbing in the anus all day like little hammers—in pile tumors and fissures. I cured a long-standing case of fissure of the anus that had resisted several operative measures and that had that prominent symptom present (throbbing as of little hammers) with one dose of Lach. Lach.

has also the same symptom in other parts of the body. Nat. mur. the same upon the inside of the skull.

Anus is sore and may be covered with little vesicles—vesicular eczema—with burning, itching in the anus at night, rawness and smarting between the nates and about the scrotum in the male.

Polyuria—colorless urine, especially at night and after hysterical spasms. Boys habitually wetting the bed as result of disease improperly treated, as measles, scarlatina, or an injury. Sulph. will be your first remedy. Urination may occur at any time during the night. Sep. urinates during *first* sleep, frequently indicated in little girls. Children who take cold easily and it settles in the head, giving them *hot* head, who also wet the bed. Bell. gives prompt relief.

A lady, who had been tormented for years, urinating in first sleep, first received Sep.—no result. When I found that she had to use constant care during her waking hours, Caust., one dose, cured. When sleeping, of course, she lost voluntary control of the sphincters, with most annoying results.

Remember the redness, panting, and excoriation of the orifices in complaints manifested in the urethra of both male and female, in gonorrhœa, and in affections of the labia. Great amount of irritation and soreness in old cases of gonorrhœal discharge. In the male, urine burns the foreskin, and may be the only symptom to be found. There is often such paucity of symptoms in these cases that the remedy is hard to find, and the patient's habits of drinking, smoking, etc., greatly hinder the search.

Desire to pass urine, coming suddenly and causing pain and great effort. Involuntary discharge of semen, with burning in the urethra, emissions brought on by self-abuse, and which have gone on to impotency. Relaxation and coldness of the penis. Sulph. produces these weaknesses, and, with few symptoms, there is no better remedy; or Sulph., Calc., Lyc. in the order named. Sep. is another good remedy, and follows them all well.

Sulph. is invaluable in balanitis, balanoposthitis; offensive and profuse sweat about the genitals, long-standing inflammation of the prostate gland, and chronic enlargement of the prostate gland. The latter will usually yield to that remedy and competes with Puls. and Staph.

Menses too late, too short duration, or suppressed. Blood thick, dark, acrid, sour smelling, making the thighs sore. Here we have the characteristic. Should a case of amenorrhœa be presented with staring eyes, burning feet, burning head, unhealthy skin, diarrhœa, driving out of bed in the morning, you can hardly help giving Sulph. In nosebleed, during menstruation, it stands next to Phos. and Lyc. Sterility with too early and too profuse menstruation. Corroding leucorrhœa of yellow mucus, preceded by pains in the abdomen. Burning in the vagina, *agg.* by hot water. Troublesome itching in the vagina, which is surrounded by pimples. Labor-like pains over the symphysis. Soreness in the vagina during coition. Vaginismus preventing coition soon after marriage—Ferr., Phos. Platina has a sense of soreness during coition. Sepia has great pain on intercourse, with soreness of vagina. Sulph. is also frequently indicated in pregnancy, and in cramping of the calves. In giving you my experience with the provings of this great remedy it is with the hope to teach you to study out new provings for yourselves. It takes practice, experience, patience, and great carefulness of detail to make use of these accumulated provings of drugs.

